# Contextual tumor suppressor function of T cell death-associated gene 8 (TDAG8) in hematological malignancies

**DOI:** 10.1186/s12967-017-1305-6

**Published:** 2017-10-10

**Authors:** Calvin R. Justus, Edward J. Sanderlin, Lixue Dong, Tianai Sun, Jen-Tsan Chi, Kvin Lertpiriyapong, Li V. Yang

**Affiliations:** 1 Division of Hematology/Oncology, Department of Internal Medicine, Brody School of Medicine, East Carolina University, 600 Moye Blvd, Greenville, NC USA; 2 Department of Anatomy and Cell Biology, Brody School of Medicine, East Carolina University, Greenville, NC 27834 USA; 3 Department of Comparative Medicine, Brody School of Medicine, East Carolina University, Greenville, NC USA; 4 Department of Molecular Genetics and Microbiology, Duke University, Durham, NC USA; 5 Center for Genomic and Computational Biology, Duke University, Durham, NC USA

**Keywords:** TDAG8, GPR65, Acidosis, Tumor microenvironment, Hematological malignancies

## Abstract

**Background:**

Extracellular acidosis is a condition found within the tumor microenvironment due to inadequate blood perfusion, hypoxia, and altered tumor cell metabolism. Acidosis has pleiotropic effects on malignant progression; therefore it is essential to understand how acidosis exerts its diverse effects. TDAG8 is a proton-sensing G-protein-coupled receptor that can be activated by extracellular acidosis.

**Methods:**

TDAG8 gene expression was analyzed by bioinformatic analyses and quantitative RT-PCR in human hematological malignancies. Retroviral transduction was used to restore TDAG8 expression in U937, Ramos and other blood cancer cells. Multiple in vitro and in vivo tumorigenesis and metastasis assays were employed to evaluate the effects of TDAG8 expression on blood cancer progression. Western blotting, immunohistochemistry and biochemical approaches were applied to elucidate the underlying mechanisms associated with the TDAG8 receptor pathway.

**Results:**

TDAG8 expression is significantly reduced in human blood cancers in comparison to normal blood cells. Severe acidosis, pH 6.4, inhibited U937 cancer cell proliferation while mild acidosis, pH 6.9, stimulated its proliferation. However, restoring TDAG8 gene expression modulated the U937 cell response to mild extracellular acidosis and physiological pH by reducing cell proliferation. Tumor xenograft experiments further revealed that restoring TDAG8 expression in U937 and Ramos cancer cells reduced tumor growth. It was also shown U937 cells with restored TDAG8 expression attached less to Matrigel, migrated slower toward a chemoattractant, and metastasized less in severe combined immunodeficient mice. These effects correlated with a reduction in c-myc oncogene expression. The mechanistic investigation indicated that Gα13/Rho signaling arbitrated the TDAG8-mediated c-myc oncogene repression in response to acidosis.

**Conclusions:**

This study provides data to support the concept that TDAG8 functions as a contextual tumor suppressor down-regulated in hematological malignancies and potentiation of the TDAG8 receptor pathway may be explored as a potential anti-tumorigenic approach in blood cancers.

**Electronic supplementary material:**

The online version of this article (doi:10.1186/s12967-017-1305-6) contains supplementary material, which is available to authorized users.

## Background

In the early twentieth century Otto Warburg recognized a metabolic phenomenon that occurred in cancer cells, currently known as the Warburg effect [1–4]. Warburg discovered cancer cells favor glycolysis rather than oxidative phosphorylation for energy production, even in the presence of oxygen [1–4]. It was originally hypothesized that irreversible injury of mitochondrial respiration is the cause of cancer cell formation [1]. However, this hypothesis was to some extent discredited as most cancers retain their ability to exploit mitochondrial respiration, although to a lesser degree than normal cells [5–7]. As a result of unrestricted glycolysis the tumor microenvironment is spatially acidic [8–10]. Extracellular acidosis has pleiotropic effects on tumor growth and cancer progression [10–14]. Tumor acidosis can stimulate cell death, reduce cell proliferation, and induce chromosomal instability of normal somatic cells and cancer cells [13–16]. In addition, tumor cells that become resistant to extracellular acidosis have been reported more malignant and invasive [17, 18]. Therefore, extracellular tumor acidosis augments cancer progression in a Darwinian manner worsening disease prognosis. As such, it is imperative to understand how tumor cells sense and respond to acidic surroundings for adequate comprehension of cancer development.

T cell death-associated gene 8 (TDAG8, also known as GPR65) is a member of the proton sensing G-protein coupled receptor family, which also includes GPR4, GPR68 (OGR1), and GPR132 (G2A). The family of G-protein coupled receptors is activated by extracellular acidosis, which illuminates a receptor signaling connection to the acidic conditions found in tumor microenvironment [19]. The human TDAG8 gene has been mapped to chromosome 14q31-32.1, a location that abnormalities associated with T cell lymphoma and leukemia are found and is primarily expressed in immune cells and leukocyte-rich tissues such as circulating peripheral leukocytes, spleen, thymus, and tonsils [20, 21]. TDAG8 has been reported to have both pro- and anti-oncogenic effects in blood cancers, which indicate TDAG8 effects are cell type and context dependent [22–24]. Therefore, it is imperative to understand the contextual effects of TDAG8 in hematological malignancies. Blood cancer cells are generally glycolytic and produce lactic acid that can acidify the microenvironment. Some types of hematological malignancies, such as lymphomas, have the ability to form solid tumors in which the tumor microenvironment is acidic. Other types of hematological malignancies, such as leukemia and multiple myeloma, form in and metastasize to bone marrow that has hypoxic and possibly acidic niches [25, 26]. In rare cases, systemic lactic acidosis occurs in patients with hematological malignancies and is associated with poor prognosis [27].

In this study the effects of acidosis and TDAG8 gene expression was investigated in blood cancers. TDAG8 gene expression was examined in hematological malignancies revealing a significant reduction in comparison to normal immune cells and leukocyte-rich tissues. Functional studies demonstrated that restoration of TDAG8 gene expression suppressed the growth, migration and metastasis of blood cancer cells and sensitized them to extracellular acidosis.

## Methods

### Bioinformatics

TDAG8 gene expression was investigated in blood cancers using the Oncomine database. A differential analysis of TDAG8 gene expression was performed between leukemia, lymphoma, and myeloma datasets and normal immune cells and leukocyte-rich tissue. Additional bioinformatics analyses were performed on datasets from the National Center for Biotechnology Information (NCBI) Gene Expression Omnibus (GEO). The raw data was downloaded using expression console software HG_u133_Plus_2 and HG_u133a_2 as libraries. The analysis was run and the RMA normalization method was used to generate the values graphed. The output file was then merged with the probe set downloaded from Affymetrix.

### Plasmid constructs

The MSCV-huTDAG8-IRES-GFP construct was made previously and the empty construct, MSCV-IRES-GFP, was used as a control [23]. For the pMSCV-huTDAG8-IRES-YFP-P2A-OF-LUC construct, the open reading frame of human TDAG8 was amplified using primers designed to contain the EcoRI and XhoI restriction enzyme sites: 5′-ATAAGAATGAA TTCACCATGAACAGCACATGTATTGAAGAA-3′ and 5′-ATAAGAATGAATTCCTCGAG CTACTCAAGGACCTCTAATTCCAT-3′. The pMSCV-IRES-YFP-P2A-OF-LUC plasmid was then digested with EcoRI and XhoI and the huTDAG8 open reading frame was cloned into it generating the pMSCV-huTDAG8-IRES-YFP-P2A-OF-LUC construct. The resultant construct was verified by DNA sequencing.

### Cell lines and culture

All the cancer cell lines were obtained from American Type Culture Collection (ATCC) with characterization as described in the product sheet. All cell lines were cultured in Roswell Park Memorial Institute medium (RPMI) supplemented with 10% fetal bovine serum (FBS) in a tissue culture incubator set at 37 °C and 5% CO_2_. RPMI/FBS medium was buffered using 7.5 mM HEPES, 7.5 mM EPPS and 7.5 mM MES, known collectively as RPMI/HEM, as previously described [23]. Cells used for experiments were > 95% viable as assessed by the trypan blue dye exclusion method.

To generate green fluorescent protein (GFP) and yellow fluorescent protein (YFP)/luciferase (Luc) cell lines with restored TDAG8 gene expression, retroviral-mediated cell transduction was performed as previously described [28]. To generate the Gα13 signaling-deficient cell lines the p115 Rho RGS construct was subcloned into the pQCXIP vector as previously described [28, 29]. The p115 Rho RGS-pQCXIP and empty pQCXIP retroviral vectors were then stably transduced into U937 cells.

### EdU cell proliferation assay

The Click-iT^®^ Plus EdU Imaging Kit (Thermo Fisher Scientific) was used to examine U937/Vector and U937/TDAG8 cell proliferation. Fluorescence microscopy with the EVOS^®^ *fl* Digital Inverted Microscope was used to take images of cells incorporating Hoescht^®^ 33,342 dye and the EdU analogue, and also images with transmitted light in the same field of view at a 200 × magnification. Adobe Photoshop’s counting tool was used to count the number of total cells according to Hoescht^®^ 33,342 dye and proliferating cells according to EdU positive cells.

### Cell growth competition assay

The same number of non-GFP expressing cells (U937/Parental or RPMI 8226/Parental) and GFP expressing cells (Vector or TDAG8) were mixed into a well and the percentages of non-GFP and GFP cells were measured using flow cytometry over 14 days. Cell population percentages were analyzed and graphed.

### Annexin V/7AAD staining

Cells were stained with the PE Annexin V Apoptosis Detection Kit I (BD Biosciences). Emission of single cell fluorescence was measured at 572 nm for Annexin V, 647 nm for 7AAD, and 525 nm for GFP. The results were analyzed with CellQuest software (BD Biosciences).

### Quantitative reverse transcription-polymerase chain reaction (RT-PCR)

Gene expression was determined by quantitative RT-PCR as previously described [23], using the following TaqMan predesigned primer/probes from Invitrogen, TDAG8 (Hs00269247_s1), c-myc (Hs00153408_m1), and β-actin (Hs99999903_m1). Relative gene expression was calculated using the 2^−ΔΔCt^ method [30].

### Western blotting

Western blot was performed as previously described [23]. Antibodies of c-myc (product #5605), phospho-paxillin (Y118) (#2541), phospho-CREB (S133) (#9198), CREB (#9197), caspase-3 (#9665), caspase-9 (#9502), cleaved PARP XP (#5625), and β-actin (#4970) were purchased from Cell Signaling Technology.

### Histology

Immunohistochemistry (IHC) for antibodies against c-myc (Abcam, #ab32072), cleaved PARP (Cell Signaling Technology, #5625), Ki67 (Abcam, #ab15580), and human nucleoli (Abcam, #ab190710) was performed on paraffin-embedded 5 µm sections. Antigen retrieval was performed by boiling slides in TRIS–EDTA + 0.1% Tween-20 pH 9.0 antigen retrieval buffer for 10–18 min. SuperPicture™ 3rd Gen IHC Detection Kit (Invitrogen) was used to complete immunohistochemistry.

### Transwell cell migration assays

Two hundred microliter of cells were seeded into the transwell insert at 5 × 10^6^ cells/ml in migration buffer consisting of RPMI media buffered to pH 7.4 and pH 6.4 without FBS and supplemented with 0.1% BSA. Chemoattractant (5 ng/mL SDF1-α) was then added into the bottom well. The plates were then incubated at 37 °C and 5% CO_2_ for 5 h. After 5 h the number of migrated cells was quantified using flow cytometry.

### Cell attachment assays

Matrigel solution was added into each well to form a thin layer of gel. U937 cells were plated onto Matrigel and incubated at 37 °C and 5% CO_2_ for 1 h in culture media buffered to pH 7.4 or 6.4. After 1 h the media was aspirated and the wells were washed 4 times with RPMI to remove non-adherent cells. For the endothelial cell attachment assay Human Umbilical Vein Endothelial cells (HUVEC) were seeded on 0.1% gelatin-coated plates to create a monolayer. Next, U937 cells were plated in each well in culture media buffered to pH 7.4 or 6.4. Plates were incubated at 37 °C and 5% CO_2_ for 1 h. After 1 h the media was aspirated and the wells were washed 4 times with RPMI. For all attachment assays several images were taken in various areas of the well to give an adequate representation. Cell attachment was quantified by counting every cell in each field of view.

### Primary tumor and metastasis xenograft

NOD.CB17-Prkdc<SCID>/J mice were purchased from Jackson Laboratories and bred at East Carolina University animal facilities for research purposes. All experimental procedures were performed according to the NIH guide for the care and use of laboratory animals and the institutional regulations. In each experiment, 5 × 10^6^ U937 or Ramos cells were injected into the flanks of SCID mice. Tumor growth was measured daily using a caliper. Tail vein injections were performed with 2 × 10^6^ U937 cells in SCID mice. When mice reached endpoint parameters, e.g. lethargy, hunched posture, and unkempt appearance, they were euthanized for analysis. The experiment was terminated 8 months after injection when the remaining mice showed no signs of disease progression. To monitor U937-Luc tumor growth in vivo, intraperitoneal injection with d-Luciferin (Caliper Life Sciences, Catalogue # 119222) at 15 mg/ml in DPBS was performed. Subsequently, mice were imaged using the IVIS Lumina XR unit.

### Statistical analysis

Statistical analysis was performed using the GraphPad Prism software. The unpaired *t* test was used to test for statistical significance between 2 groups. Error bars represent ± standard error (SEM) in all graphs. ns: P > 0.05, *P < 0.05, **P < 0.01, ***P < 0.001.

## Results

### TDAG8 gene expression is reduced significantly in hematologic malignancies in comparison to normal immune cells and leukocyte-rich tissue

**Fig. 1:**
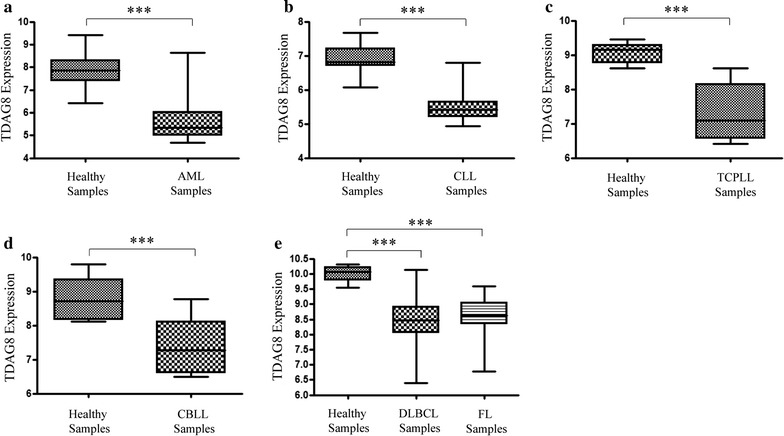
TDAG8 gene expression is reduced in patients with blood cancers in comparison to healthy donor’s immune cells or leukocyte-rich tissue. **a** The expression of TDAG8 is reduced 4.5-fold in CD34+ bone marrow and CD34+ peripheral blood cells isolated from patients with AML (N = 20) in comparison to CD34+ bone marrow and CD34+ peripheral blood cells isolated from healthy donors (N = 26). **b** The expression of TDAG8 is reduced 2.9-fold in CD19+/CD5+ peripheral blood mononuclear cells isolated from patients with CLL (N = 11) in comparison to CD19+/CD5+ peripheral blood mononuclear cells isolated from healthy donors (N = 39). **c** The expression of TDAG8 is reduced 3.6-fold CD3+ peripheral blood cells (N = 8) isolated from patients with TCPLL in comparison to CD3+ peripheral blood cells isolated from healthy donors (N = 6). **d** The expression of TDAG8 is reduced 2.7-fold in CD19+ peripheral blood lymphocytes isolated from patients with CBLL (N = 6) in comparison to CD19+ peripheral blood lymphocytes isolated from healthy donors (N = 11). **e** The expression of TDAG8 is reduced by 3.0-fold in DLBCL biopsy samples (N = 73) in comparison to B-lymphocytes isolated from healthy donors (N = 9). The expression of TDAG8 is also reduced by 2.8-fold in human FL biopsy samples (N = 38) in comparison to B-lymphocytes isolated from healthy donors (N = 9). *AML* acute myeloid leukemia, *CLL* chronic lymphocytic leukemia, *DLBCL* diffuse large B-cell lymphoma, *FL* follicular lymphoma, *TCPLL* T-cell prolymphocytic leukemia with inv(14)(q11q32), *CBLL* chronic B-cell lymphocytic leukemia. Y-axis is Log_2_ scale. ***P < 0.001

**Fig. 2:**
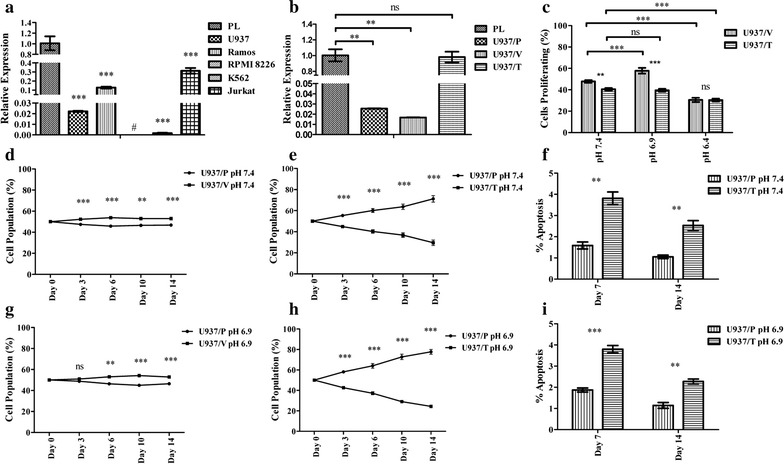
Restoration of TDAG8 gene expression in U937 cells provides a growth disadvantage at physiological pH 7.4 and mildly acidic pH 6.9. **a** Quantitative RT-PCR demonstrates a reduction of TDAG8 gene expression in U937, ramos, RPMI 8226, K562, and Jurkat cells in comparison to peripheral leukocytes pooled from 426 healthy donors. **b** Quantitative RT-PCR demonstrates restoration of TDAG8 gene expression in U937 acute myeloid leukemia cells following transduction and cell sorting. **c** Mild acidosis (pH 6.9) increases U937/Vector cell proliferation while severe acidosis (pH 6.4) inhibits cell proliferation. Restoring TDAG8 gene expression in U937 cells inhibits cell proliferation in comparison to U937/Vector cells. **d** The expression of the empty MSCV-IRES-GFP construct in U937 cells does not substantially affect the U937/Vector cell population percentage in comparison to U937/Parental cells at pH 7.4 from day 0 to day 14. **e** The restoration of TDAG8 gene expression in U937 cells reduces cell growth in comparison to U937/Parental cells at physiological pH 7.4. **f** Restoring TDAG8 gene expression in U937 cells increases apoptosis throughout the cell competition assay at pH 7.4. **g** The expression of the empty MSCV-IRES-GFP construct in U937 cells does not substantially affect the U937/Vector cell population percentage in comparison to U937/Parental cells at pH 6.9 from day 0 to day 14. **h** The difference between the U937/TDAG8 cell population and the U937/Parental cell population throughout the competition assay at acidic pH 6.9 is greater in comparison to pH 7.4 treatment. **i** Restoring TDAG8 gene expression in U937 cells increases apoptosis throughout the cell competition assay at pH 6.9. Pooled RNA from 426 normal peripheral leukocytes was purchased from Clontech Laboratories, Inc. to be used as a control for a healthy immune cell comparison. ns: P > 0.05, *P < 0.05, **P < 0.01, ***P < 0.001

Oncomine bioinformatics analysis identified that TDAG8 gene expression was reduced in multiple forms of blood cancer, including acute myeloid leukemia (AML) (2.2 and 6.1-fold) [31, 32], chronic lymphocytic leukemia (CLL) (3.3-, 1.7-, 2.3-, and 2.6-fold) (Table 1) [31, 33–35], T-cell acute lymphoblastic leukemia (TCALL) (3.1-fold), B-cell childhood acute lymphoblastic leukemia (BCCALL) (2.5-fold), B-cell acute lymphoblastic leukemia (BALL) (2.4-fold), pro B-cell acute lymphoblastic leukemia (PBALL) (1.3-fold), hairy cell leukemia (HCl) (1.9-fold), and T-cell prolymphocytic leukemia (TCPLL) (3.9-fold) (Table 1) [31, 33, 36]. In addition, TDAG8 gene expression is reduced in several lymphomas such as diffuse large B-cell lymphoma (DLBCL) (1.4- and 1.9-fold) [33, 34], follicular lymphoma (FL) (1.4- and 2.0-fold) [33, 34], pleural effusion lymphoma (PEL) (2.7-fold) [33], and Burkitt lymphoma (BL) (1.8-fold) [33] (Table 1). Lastly, TDAG8 expression was found reduced in smoldering myeloma (SM) (2.6-fold) (Table 1) [37]. The search in the Oncomine Research Premium Edition also revealed various cancer cell lines that were resistant to chemotherapeutics had reduced TDAG8 gene expression in comparison to cancer cell lines that were sensitive to those particular chemotherapeutics (Table 2) [38, 39]. A separate analyses of datasets from NCBI GEO revealed that TDAG8 gene expression is significantly reduced 4.5-fold in AML (GEO Series ID GSE9476), 2.9-fold in CLL (GEO Series ID GSE22529), 3.6-fold in TCPLL with inv(14)(q11q32) (GEO Series ID GSE5788), 2.7-fold in chronic B-cell lymphocytic leukemia (CBLL) (GSE26725), 3.0-fold in DLBCL (GEO series ID GSE12195), and 2.8-fold in FL (GEO series ID GSE12195) (Fig. 1a–e). The same pattern was found in various blood cancer cell lines. For example, TDAG8 gene expression was reduced 45-fold in U937 cells, 8-fold in Ramos cells, undetectable in RPMI 8226 cells, 625-fold in K562 cells, and 3-fold in Jurkat cells in comparison to normal peripheral blood leukocytes (Fig. 2a).

**Table 1:** TDAG8 gene expression is reduced significantly in blood cancers

	Leukemia						
Source:	Rose et al.			Hafer et al.			
Cancer	CLL	TCALL	BCCALL	BALL	AML	CLL	PBALL
Fold change	− 3.25	− 3.13	− 2.497	− 2.372	− 2.236	− 1.706	− 1.317
P	2.3E^−7^	1.6E^−40^	8.1E^−32^	1.1E^−26^	1.6E^−27^	1.8E^−16^	1.6E^−4^
N	38	174	359	147	542	448	70

	Leukemia						
Source:	Basso et al.	Aliz et al.	Stegmaier et al.	Durig et al.			
Cancer	CLL	HCl	CLL	AML	TCPLL		
Fold change	− 2.251	− 1.917	− 2.642	− 6.115	− 3.902		
P	8.1E^−10^	1.6E^−5^	3.4E^−5^	1.0E^−3^	1.0E^−3^		
N	12	16	12	9	6		

	Lymphoma						Myeloma
Source:	Rose et al.			Basso et al.			Zhan et al.
Cancer	DLBCL	FL	PEL	BL	FL	DLBCL	SM
Fold change	− 1.439	− 1.354	− 2.687	− 1.761	− 2.021	− 1.874	− 2.628
P	1.5E^−2^	3.0E^−2^	1.0E^−3^	1.5E^−4^	1.5E^−6^	1.3E^−6^	1.9E^−2^
N	38	7	9	17	6	32	12

*CLL* chronic lymphocytic leukemia, *TCALL* T-cell acute lymphoblastic leukemia, *BCCALL* B-cell childhood acute lymphoblastic leukemia, *BALL* B-cell acute lymphoblastic leukemia, *AML* acute myeloid leukemia, *PBALL* pro B-cell acute lymphoblastic leukemia, *HCl* hairy cell leukemia, *TCPLL* T-cell prolymphocytic leukemia. TDAG8 gene expression is also reduced significantly in *DLBCL* diffuse large B-cell lymphoma, *FL* follicular lymphoma, *PEL* pleural effusion lymphoma, *BL* Burkitt’s lymphoma, *SM* smoldering myeloma. Rose et al. normal blood cell control: B-lymphocyte (4), CD4+ T-lymphocyte (5), germinal center B-lymphocyte (1), umbilical cord blood B-lymphocyte (1), umbilical cord blood T-lymphocyte (1). Hafer et al. normal blood cell control: peripheral blood mononuclear cell (74). Basso et al. normal blood cell control: B-lymphocyte (5), centroblast (5), memory B-lymphocyte (5), naive pre-germinal center B-lymphocyte (5), small cleaved follicle center cell (5). Aliz et al. normal blood cell control: B-lymphocyte (16), CD4+ T-lymphocyte (6), centroblast (1), germinal center B-lymphocyte (2), lymph node (1), memory B-lymphocyte (3), tonsil (1). Stegmaier et al. normal blood cell control: monocyte (3), neutrophil (3). Durig et al. normal blood cell control: CD3+ peripheral blood cell (8). Zhan et al. normal blood cell control: bone marrow (22)

**Table 2:** Reduced TDAG8 gene expression correlates with resistance to various chemotherapeutics

Drug	Elesclomol (leukemia)	Panobinostat (multi-cancer)	Irinotecan (multi-cancer)	Topotecan (multi-cancer)	Navitoclax (leukemia)	MK2206 (leukemia)
Mechanism of action	Unknown oxidative Stress	Histone deacetylase inhibitor	Topoisomerase inhibitor	Topoisomerase inhibitor	Bcl-2, Bcl-XL, and Bcl-w inhibitor	AKT inhibitor
Resistant cell lines (R*n*)	21	164	84	124	12	17
Sensitive cell lines (S*n*)	4	86	71	154	6	3
TDAG8 gene expression of S*n* vs R*n*	+ 2.3	+ 2.7	+ 3.2	+ 2.2	+ 2.4	+ 3.8
P	4.8E^−2^	2.6E^−7^	4.2E^−8^	2.9E^−10^	3.4E^−2^	7.8E^−4^

Cancer cells of various origins categorized as resistant to specific chemotherapeutics demonstrate a significant reduction in TDAG8 gene expression. The expression of TDAG8 in 4 cell lines sensitive to elesclomol is 2.3-fold higher than in 21 cell lines resistant. The expression of TDAG8 in 86 cell lines categorized as sensitive to panobinostat is 2.7-fold higher than in 164 cell lines categorized as resistant. The expression of TDAG8 in 71 cell lines categorized as sensitive to irinotecan is 3.2-fold higher than in 84 cell lines categorized as resistant. The expression of TDAG8 in 154 cell lines categorized as sensitive to topotecan is 2.2-fold higher than in 124 cell lines categorized as resistant. The expression of TDAG8 in 6 cell lines categorized as sensitive to navitoclax is 2.4-fold higher than in 12 cell lines categorized as resistant. The expression of TDAG8 in 3 cell lines categorized as sensitive to MK2206 is 3.8-fold higher than in 17 cell lines categorized as resistant

### TDAG8 gene expression provides a disadvantage to blood cancer cell growth

As TDAG8 gene expression is commonly downregulated in hematological malignancies (Tables 1, 2 and Fig. 1), we investigated its function by restoring TDAG8 expression in blood cancer cell lines such as U937, Ramos and RPMI 8226 with low TDAG8 expression. The U937 cell line was originally established from a patient with histiocytic lymphoma and later found to possess characteristics of acute myeloid leukemia (AML) cells [40]. U937 cells were used as a model system for this study because the cells have features of both lymphoma and leukemia. Proliferation of U937 cells transduced with a construct containing the human TDAG8 gene (U937/TDAG8) was compared to U937 cells transduced with an empty vector (U937/Vector). The expression level of TDAG8 mRNA in U937/TDAG8 cells was restored to the similar level as that in normal leukocytes (Fig. 2b). Due to the lack of a reliable TDAG8 antibody, we were unable to detect TDAG8 protein expression. Instead, we examined the TDAG8 downstream signaling activities and found the phosphorylation of CREB was higher in U937/TDAG8 cells than that in U937/Vector cells (Additional file 1: Figure S1A), suggesting that restoration of TDAG8 expression augments its downstream G protein signaling. Similarly, CREB phosphorylation was increased in Ramos/TDAG8 cells with the restoration of TDAG8 expression (Additional file 1: Figure S1B, C).

In comparison to U937/Vector cells, U937/TDAG8 cells proliferated 7% less (47.8% vs. 40.5% proliferating cells) at physiological pH 7.4 (Fig. 2c). When U937/Vector cells were treated with pH 6.9, there was a 10% increase in cell proliferation in comparison to physiological pH 7.4. However, restoring TDAG8 gene expression sensitized U937 cells to media buffered to pH 6.9 and suppressed cell proliferation at pH 6.9 (Fig. 2c). Severe acidosis, pH 6.4, significantly reduced cell proliferation in both U937/Vector and U937/TDAG8 cells (Fig. 2c).

Next, cell growth competition assays were performed to demonstrate negative selection of U937 cells expressing the TDAG8 gene. In the cell growth competition assay there was a slight change in the U937/Parental-U937/Vector cell populations (50 ± 5%) after 14 days (Fig. 2d). However, the U937/TDAG8 cell population was only 30% of the total population of cells in comparison to 70% of the U937/Parental cells at pH 7.4 (Fig. 2e). Quantitative RT-PCR also demonstrated that restoration of TDAG8 expression in U937/TDAG8 cells reduced the expression of c-myc oncogene on day 0 and day 14 of the competition assay in comparison to the U937/Vector cells (Additional file 1: Figure S2A, B). The growth competition assay was also performed in medium buffered to mildly acidic pH 6.9. The U937/TDAG8 cell population was significantly reduced to 23% of the total population of cells at pH 6.9 (Fig. 2h). The U937/Vector and U937/Parental cells showed slight changes (50 ± 5%) relative to each other at pH 6.9 (Fig. 2g). Results were similar when the competition assay was performed with the RPMI 8226/Vector and RPMI 8226/TDAG8 myeloma cells (Additional file 1: Figure S3).

The GFP mean fluorescence value remained stable from day 0 to day 14 with little variation in the U937/Vector cells when co-cultured with the U937/Parental cells (Additional file 1: Figure S2C). However, U937/TDAG8 cell GFP mean fluorescence value as an indicator of TDAG8 transgene expression was considerably reduced (Additional file 1: Figure S2C). When comparing day 0 with day 14 at pH 6.9 there was a 65% reduction in GFP mean fluorescence value of U937/TDAG8 cells (Additional file 1: Figure S2D).

Annexin V PE/7AAD staining was performed at day 7 and day 14 of the competition assay to examine U937 cell apoptosis. It was determined there was a significant increase of apoptosis in U937/TDAG8 cells in comparison to U937/Parental cells at pH 7.4 and at pH 6.9 (Fig. 2f, i). Increased apoptosis of U937/TDAG8 cells also correlated with an increase in caspase 3, caspase 9, and PARP cleavage (Additional file 1: Figure S4).

### Restoration of TDAG8 gene expression reduces primary tumor growth in severe combined immunodeficient (SCID) mice

**Fig. 3:**
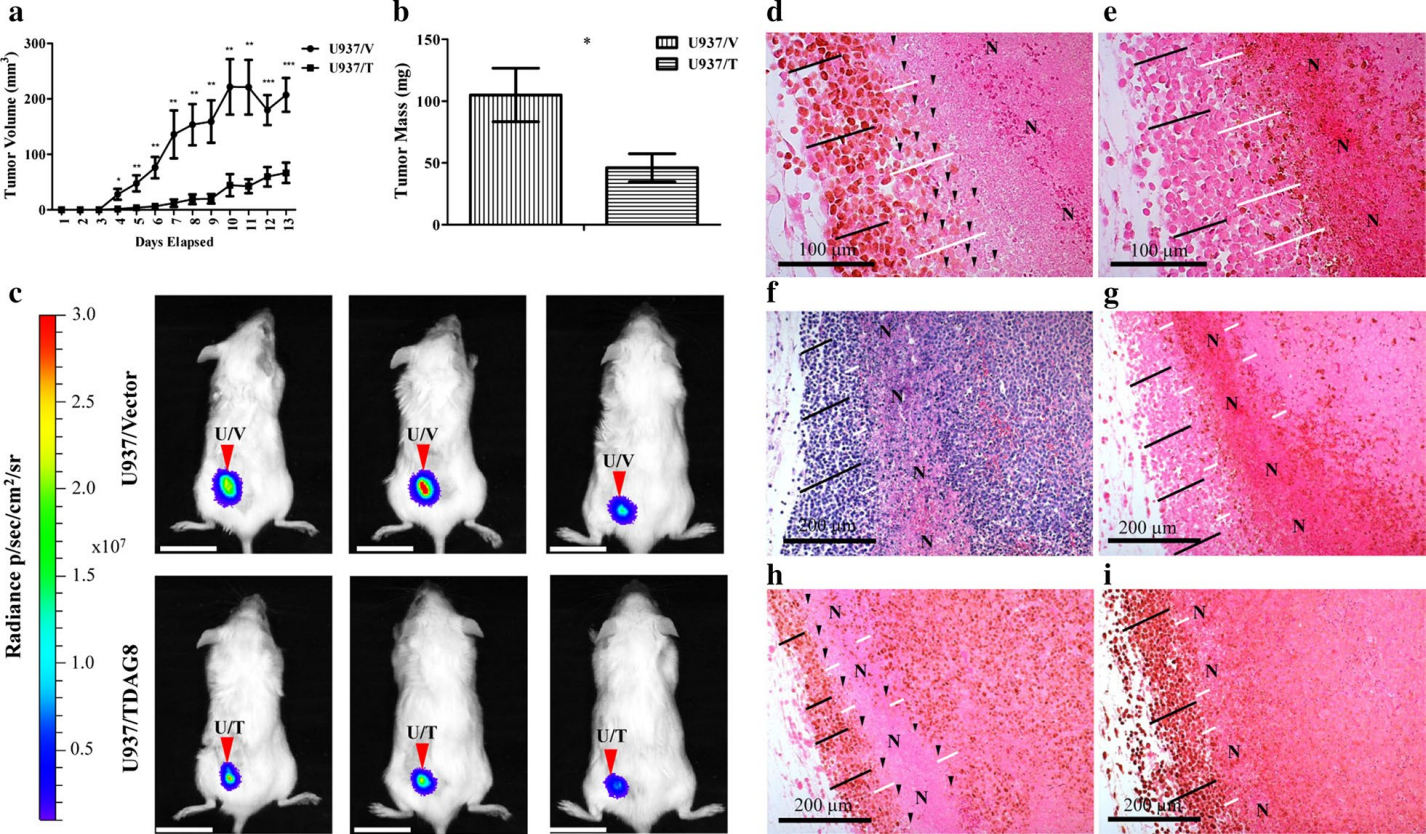
Restoring TDAG8 gene expression in U937 cells reduces primary tumor growth in SCID mice. **a** U937/TDAG8 tumor growth was significantly reduced in comparison to U937/Vector tumors from day 4 to necropsy (N = 14). **b** U937/TDAG8 tumor mass was reduced significantly in comparison to U937/Vector tumors following necropsy. **c** In vivo imaging of 3 mice injected with U937/Vector-Luc or U937/TDAG8-Luc cells 6 days after injection. Scale Bar = 2 cm. **d**, **e** Immunohistochemistry of c-myc **d** and cleaved PARP **e** in U937/Vector tumors demonstrating regions nearest necrotic areas display reduced c-myc oncogene expression while still live. **f**–**i** Hematoxylin and eosin staining (**f**) and immunohistochemistry of U937/Vector tumors demonstrating invasive peripheral regions of U937 tumors display increased proliferation by c-myc (**h**) and Ki67 (**i**) while demonstrating less apoptosis, cleaved PARP (**g**). *N* necrotic. White lines indicate areas adjacent to necrotic zones. Black lines indicate tumor cells that are invasive correlating with higher c-myc and Ki67 expression. Black arrowheads indicate single tumor cells that demonstrate reduced or no c-myc expression. *P < 0.05, **P < 0.01, ***P < 0.001

U937 cells were originally isolated from a patient with histiocytic lymphoma and can form solid tumors, which are known to be spatially acidic. To determine the effects of restoring TDAG8 gene expression on U937 primary tumor growth, subcutaneous xenograft experiments were performed in SCID mice. A significant inhibition of U937/TDAG8 tumor growth was observed throughout the experiments (Fig. 3a). In addition, overall tumor mass was reduced by ~ 50% in U937/TDAG8 tumors (Fig. 3b). Similar trends were found following Ramos/Vector and Ramos/TDAG8 lymphoma cell injections into SCID mice (Additional file 1: Figure S5). In vivo luminescence imaging was also used to visualize tumor growth over 2 weeks. The pattern previously observed in U937/TDAG8 cells, i.e. reduced tumor growth, was consistent with the U937/TDAG8-Luc cells expressing TDAG8 and the luciferase marker gene (Fig. 3c).

### Decreased c-myc expression is detected in the tumor zone close to necrotic regions

Immunohistochemistry (IHC) of c-myc expression revealed that sections nearest necrotic regions of tumors, known to be acidic [17], had reduced expression of c-myc (Fig. 3d–i). The areas that were investigated for low c-myc expression were negative for cleaved PARP indicating that intact cells nearest necrotic regions were viable (Fig. 3e, g). In addition, it was also revealed that invasive cancer cells in the tumor peripheral regions had increased expression of c-myc and Ki-67 and were negative for cleaved PARP (Fig. 3g–i).

### Acidosis/TDAG8 reduces c-myc expression through Gα13/Rho signaling

**Fig. 4:**
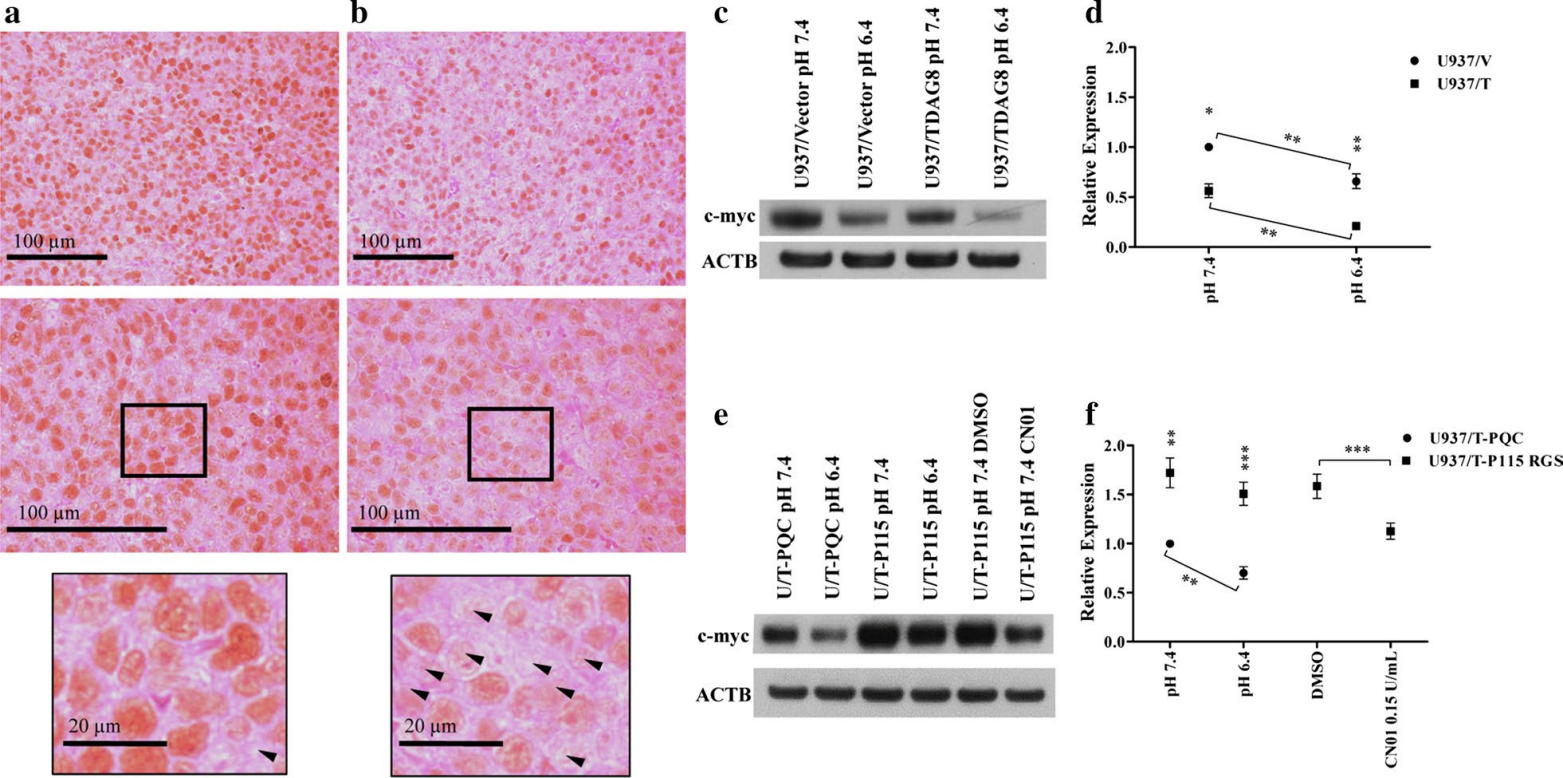
Restoration of TDAG8 gene expression reduces c-myc oncogene expression through Gα13/Rho signaling. **a** IHC demonstrates the majority of cells within the U937/Vector tumors away from necrotic or heterotypic areas are positive for c-myc expression as indicated by DAB chromogen (N = 5). **b** There were less cells within the U937/TDAG8 tumors away from necrotic or heterotypic areas positive for c-myc expression as indicated by DAB chromogen. Arrowheads indicate cells not expressing c-myc. Observed in at least 3, 5 µm sections of 5 different tumors for each experimental group. **c** Restoration of TDAG8 in U937 cells reduces c-myc expression in response to treatment with media buffered to physiological pH 7.4 and acidic pH 6.4. **d** Quantification of c-myc Western blot data displaying the significant inhibition of c-myc mediated by acidosis and TDAG8 gene expression. **e**, **f** Gα13/Rho signaling is responsible for a TDAG8 mediated inhibition of c-myc expression. *P < 0.05, **P < 0.01, ***P < 0.001

IHC analysis of c-myc protein expression revealed that U937/TDAG8 tumor cells expressed lower levels of c-myc in comparison to U937/Vector control (Fig. 4a, b). It was also qualitatively determined that c-myc expression was undetectable in numerous U937/TDAG8 cells in the tumor (Fig. 4b). In comparison, the majority of U937/Vector cells expressed c-myc at a high level (Fig. 4a). It was also determined that restoring TDAG8 gene expression reduces c-myc protein expression significantly in comparison to the U937/Vector control cells at physiological pH 7.4 in vitro (Fig. 4c, d). In addition, activation of TDAG8 by extracellular acidification, representing similar conditions as the tumor microenvironment, further reduces c-myc expression in comparison to pH 7.4 treatment and to the U937/Vector controls (Fig. 4c, d). These observations are consistent with previous studies showing that TDAG8 has some constitutive activities at the physiological pH and is fully activated under acidic pH [41].

Following inhibition of Gα13 signaling by the p115 RGS construct [28, 29], Western blot analysis determined that c-myc expression was restored in the U937/TDAG8 cells after treatment with media buffered to pH 7.4 and pH 6.4 (Fig. 4e, f). These results reveal that Gα13 signaling is central for TDAG8-mediated c-myc inhibition at physiological pH 7.4 and acidic pH 6.4. The activation of Rho GTPases with CN01, downstream from the p115-RGS Gα13 inhibition, reduced the expression of c-myc, indicating that Gα13/Rho signaling in U937 cells inhibits c-myc expression (Fig. 4e, f). Similar results were found for caspase 3, caspase 9, and PARP cleavage following inhibition of Gα13 signaling and activation of Rho (Additional file 1: Figure S6). Therefore, the data suggests TDAG8 exerts its growth inhibitory effects through activation of Gα13/Rho signaling in U937 cells.

### Restoration of TDAG8 gene expression in U937 cells reduces cell attachment and migration correlating with altered focal adhesion dynamics

**Fig. 5:**
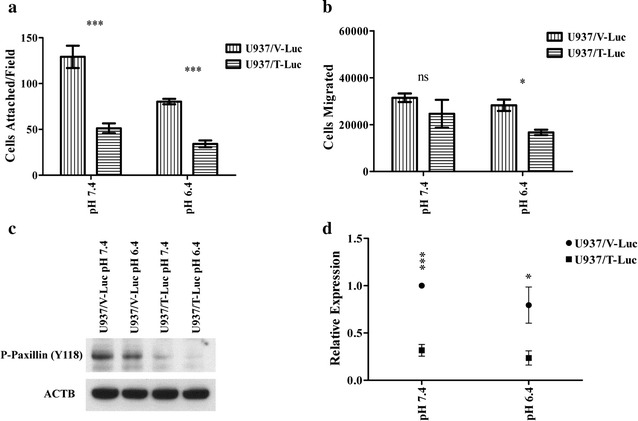
TDAG8 gene expression reduces U937 cell attachment and migration. **a** Restoration of TDAG8 gene expression inhibits U937 cell attachment to matrigel. **b** U937 cell migration towards SDF1-α is inhibited by TDAG8 gene expression at pH 7.4 and pH 6.4. **c** Acidosis and restoration of TDAG8 gene expression reduces phosphorylation of paxillin at Y118 in U937 cells. **d** Quantification of paxillin phosphorylation at Y118 reveals a significant reduction in U937/TDAG8-luc cells at pH 7.4 and 6.4. ns: P > 0.05, *P < 0.05, P < 0.01, ***P < 0.001

Restoration of TDAG8 gene expression reduced the ability for U937 cells to attach to Matrigel at physiological pH 7.4 by more than 50% (Fig. 5a). Activation of TDAG8 by extracellular acidosis reduced U937 cell attachment even further demonstrating the anti-metastatic capabilities of TDAG8 (Fig. 5a). U937 cell attachment to a HUVEC monolayer was also investigated revealing a reduction in U937/TDAG8 cell attachment to endothelial cells at physiological pH 7.4 and acidic pH 6.4 (Additional file 1: Figure S7A). However, overall cell attachment to a HUVEC monolayer was increased at acidic pH 6.4 likely due to the effects of acidosis on endothelial cell-mediated attachment [42]. It was also determined that U937/TDAG8-Luc cells exhibited less cell migration towards the chemoattractant SDF-1α in comparison to U937/Vector-Luc cells (Fig. 5b). In addition, treatment with pH 6.4 further reduced the migration of U937/TDAG8-Luc cells in comparison to the U937/Vector-Luc cells (Fig. 5b). This was also observed in the U937 cells that were transduced with the GFP construct (Additional file 1: Figure S7B).

Increased activity of focal adhesion proteins such as focal adhesion kinase (FAK) and paxillin have been correlated with poor patient survival and increased malignancy of blood and other cancers [43–48]. We found that restoring TDAG8 gene expression in U937 cells significantly inhibited the phosphorylation of paxillin Y118 at pH 7.4 and pH 6.4 (Fig. 5c, d), suggesting a reduced activity of the FAK/paxillin pathway [49, 50]. In addition, when treated with media buffered to pH 6.4 the phosphorylation of paxillin Y118 was reduced in comparison to the pH 7.4 treatment groups (Fig. 5c, d).

### Restoration of TDAG8 gene expression in U937 cells reduces metastasis in SCID mice following tail vein injections

**Fig. 6:**
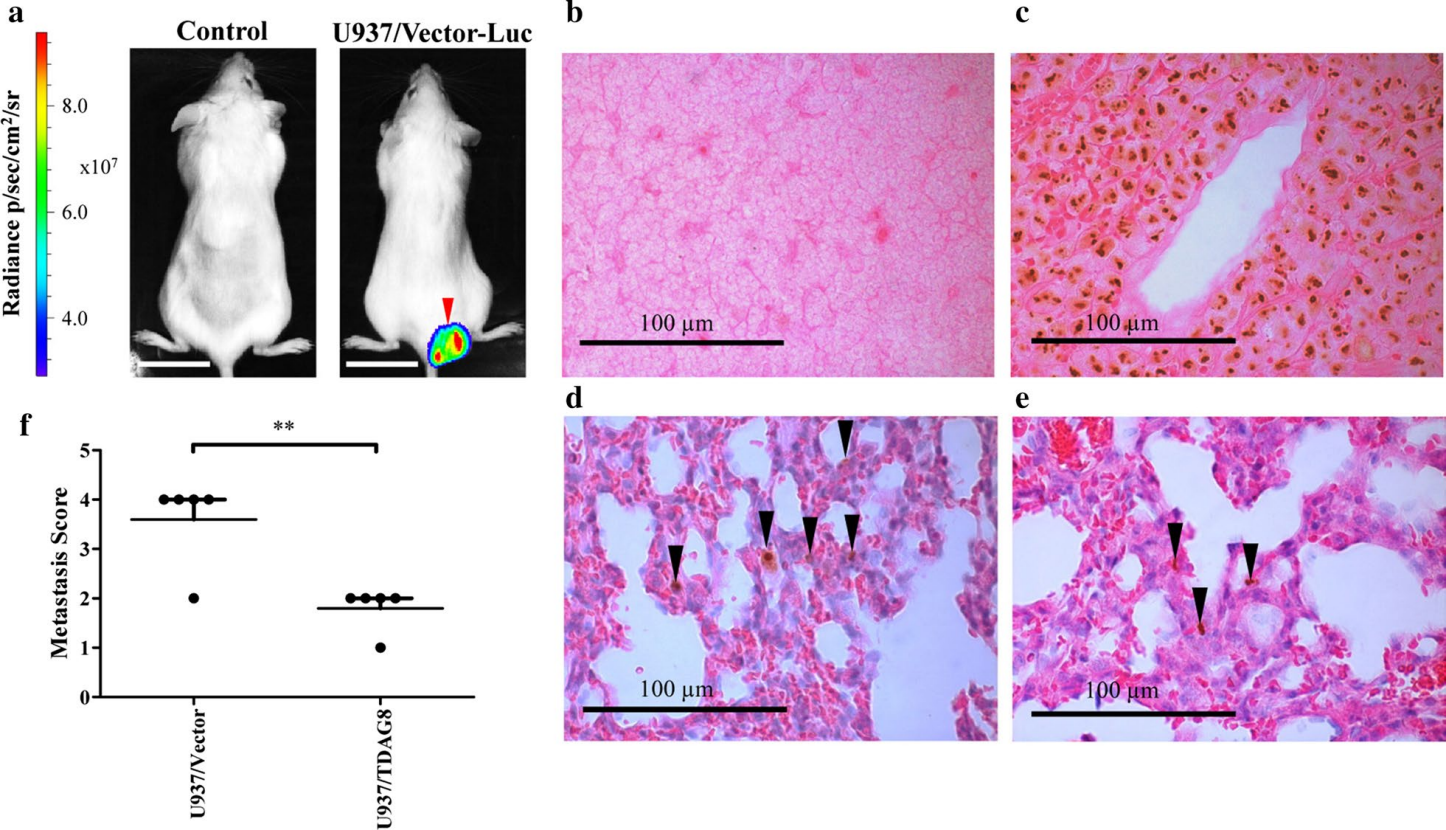
Restoration of TDAG8 gene expression reduces U937 cell metastasis in SCID mice. In vivo luminescence imaging identified larger tumors but was not sensitive enough to identify micro metastasis in SCID mice. **a** IVIS luminescence imaging. Scale bar = 2 cm. **b** Normal mouse spleen section with negative staining demonstrating the specificity of the anti-human nucleoli antibody. **c** Tumor metastasis in the lymph node was positive for human nucleoli. **d**, **e** Hematoxylin and eosin staining and IHC of the human nucleoli antibody was used to identify nuclear localization of the antibody signals of U937/Vector tumor cells in the lung of SCID mice. **f** Analysis of SCID mice lungs following IHC of human nucleoli reveals a significant reduction in metastasis. Mice with no metastasis detected were given a score of 0. Mice with < 2 average metastatic lesions in each 5 µm section of the lung were given a score of 1, from 2 to 5 average metastatic lesions were given a score of 2, from 6 to 10 average metastatic lesions were given a score of 3, and from 11 or more average metastatic lesions were given a score of 4. In addition, lesions that were larger than 20 cells or large enough to see with the IVIS Lumina XR unit was given a 4. For each experimental group injected with either U937/Vector-Luc or U937/TDAG8-Luc cells the individual scores were added up and averaged to give a metastasis score. The scoring system consisted of analyzing a total of 6 sections of lung for each mouse injected. Every section was separated by at least 20 µm of tissue to give representative sections of various areas of the lung. **P < 0.01

Overall, the in vivo imaging of SCID mice was not sensitive enough to visualize micro-metastasis of tumor cells (Fig. 6a–e). However, larger nodules and tumors were easily visualized (Fig. 6a). Metastasis of U937/Vector cells to the lymph node, bone of the limb, and other organs in SCID mice was detected by IVIS luminescence imaging (Fig. 6a; Additional file 1: Figure S7C). To further quantify metastasis, the mouse lungs were analyzed using an anti-human nucleoli antibody specific for human cells (Fig. 6b–e). The results indicate the severity of U937 cell metastasis to the lung of SCID mice was significantly reduced by restoring TDAG8 expression (Fig. 6f).

## Discussion

The effect of extracellular acidosis on cancer progression is complex; therefore it is important to understand the cancer cell biological response to it [13]. In this study it was determined that diverse extremities of extracellular acidosis had differential effects on cellular proliferation (Fig. 2c–e, g, h). It was clearly demonstrated that mild extracellular acidosis, pH 6.9, increased U937 cell proliferation while severe acidosis, pH 6.4, repressed it (Fig. 2c). In addition, this report evaluated the effects of tumor acidosis on c-myc oncogene expression in vivo. In tumor sections nearest necrotic regions, known to be acidic [17], c-myc oncogene expression was reduced significantly in live tumor cells (Fig. 3d–i). However, invasive cancer cells that were invading new tissue, devoid of blood vessels and nutrients, did not follow this pattern (Fig. 3d–i). Conversely, invasive tumor cells had increased expression of c-myc and Ki-67 signifying they are actively proliferating and invasive despite extracellular acidosis and nutrient deprivation (Fig. 3d–i). The discovered pattern of tumor cell invasiveness in harsh extracellular conditions concurrently associating with increased cell proliferative markers is similar to an acid resistant phenotype described previously [17, 18]. Importantly, understanding that extracellular acidosis reduces c-myc oncogene expression in some tumor cells while not in others provides an original understanding for the heterotypic and spatial tumor cell response to extracellular acidosis. To understand how tumor cells sense extracellular acidosis the proton sensing G-protein coupled receptor TDAG8 was investigated.

The data provided in this study indicates that TDAG8 gene expression is suppressive for U937 cell malignancy. It was discovered that TDAG8 gene expression is reduced in the majority of blood cancers in comparison to normal immune cells or leukocyte-rich tissue (Table 1 and Fig. 1). In addition, higher expression of the TDAG8 gene correlates with increased sensitivity toward various chemotherapeutics (Table 2). Using U937 acute myeloid leukemia cells as a model system TDAG8 gene expression was restored to a normal physiological level to test the hypothesis that TDAG8 gene expression provides a disadvantage for blood cancer cell malignancy. Restoring TDAG8 gene expression in U937 cells reduced cell proliferation in vitro and tumor growth and metastasis in vivo (Figs. 2, 3, 6). The ability for TDAG8 gene expression to provide a disadvantage for U937 cell proliferation, tumor growth, and metastasis confirmed that TDAG8 gene expression provides a disadvantage for blood cancer progression. Similar tumor suppressive functions of TDAG8 were observed in other blood cancer cells such as Ramos Burkitt lymphoma and RPMI 8226 multiple myeloma cells (Additional file 1: Figures S3 and S5). Moreover, TDAG8’s inherent ability to reduce c-myc expression at physiological pH 7.4 and acidic pH 6.4 is central (Fig. 4a–c) [23]. This is consistent with previous results demonstrating that TDAG8 exhibits constitutive activity at physiological pH and is further activated at acidic pH [41]. Moreover, the results from this report indicate that the inhibitory effects of acidosis on c-myc oncogene expression are partially due to TDAG8-mediated Gα13/Rho signaling (Fig. 4e–f). This idea aligns with recent reports that demonstrate Gα13/Rho signaling suppresses oncogenesis and acts as a tumor suppressor in lymphoma [51–53]. Overall, it is hypothesized in this report that extracellular acidosis provides a selective pressure against cancer cells, which modulates clonal cell evolution. Moreover, TDAG8 is a proton sensor that plays an important role in this process, which has important implications for blood cancer progression as well as cancer cell clonal evolution parallel to extracellular acidosis found within the tumor microenvironment.

TDAG8 has been reported to have a diverse repertoire of pro- and anti-oncogenic effects that are cancer type and context dependent [22–24, 54–57]. These seemingly conflicting observations can potentially be explained by TDAG8 downstream signaling—the Gα13 G-protein/Rho GTPase signaling. Studies have shown that Gα13 and Rho GTPases can have pro-oncogenic or anti-oncogenic effects in a cancer type and context dependent manner [51–53, 58–61]. Gα13 and Rho GTPases exhibit pro-tumorigenic effects in various types of epithelial cancers [58–60], but have anti-tumorigenic effects in hematological malignancies [51–53]. The differential effects of Gα13/Rho GTPases may contribute to the anti-oncogenic effects of TDAG8 in hematological malignancies and the pro-oncogenic effects in other cancer types [22–24, 54–57]. To better understand the involvement of TDAG8 in various tumor types, we performed additional bioinformatic analyses of the Oncomine database to evaluate TDAG8 (GPR65) expression in cancerous and normal tissues [62–69]. The results show that TDAG8 is over-expressed in several types of epithelial tumors, including brain tumor, kidney cancer, and head and neck cancer when compared to their normal tissue counterparts (Additional file 2: Table S1). Methodologically, however, cautions should be taken when interpreting the expression profile of TDAG8 in epithelial tumors. RNA for gene expression analysis was isolated from whole tumor tissues [62–69]. As a result, it is unclear whether the over-expression of TDAG8 is derived from epithelial cancer cells or from infiltrated leukocytes which are known to highly express TDAG8 [20, 21, 70, 71]. Also, interestingly, TDAG8 expression is either not changed [54] or down-regulated in lung cancer samples when compared to normal lung tissues (Additional file 2: Table S1). Taken together, the expression pattern of TDAG8 in epithelial tumors varies, probably complicated by infiltrated leukocytes that highly express TDAG8 [20, 21, 70, 71]. In contrast, TDAG8 expression is consistently down-regulated in hematological malignancies when compared to normal blood cells and tissues (Table 1 and Fig. 1). Moreover, functional results from this study, together with previous studies [22, 23], suggest that TDAG8 acts as a contextual tumor suppressor in hematological malignancies.

## Conclusions

In summary, this study reveals that the acid-sensing receptor TDAG8 (GPR65) is down-regulated in multiple types of hematological malignancies, including leukemia, lymphoma and multiple myeloma. Our in vitro and in vivo results demonstrate that TDAG8 functions as a tumor suppressor in hematological malignancies and sensitizes blood cancer cells to acidotic stress in the microenvironment. Mechanistically, the TDAG8-mediated Gα13/Rho signaling results in the reduction of c-myc oncogene expression which may contribute to TDAG8 tumor suppressive effects. This observation is also concordant with the tumor suppressor function of Gα13/Rho in hematological malignancies [51–53]. Our findings in this study suggest that potentiation of the TDAG8 (GPR65) receptor pathway may be exploited as a potential therapeutic approach for the treatment of hematological malignancies.

## Additional files

**Additional file 1: Figure S1.** Restoration of TDAG8 gene expression results in increased phosphorylation of CREB at serine 133 in U937 and Ramos cells. (A) Restoration of TDAG8 gene expression in U937 cells results in stimulation of CREB phosphorylation at serine 133 indicating the TDAG8 activity level is increased. (B) TDAG8 gene expression is restored in Ramos cells to a level that is physiologically relevant. (C) Restoration of TDAG8 gene expression in Ramos cells results in stimulation of CREB phosphorylation at serine 133 indicating the TDAG8 activity level is increased. **P < 0.01. **Figure S2.** TDAG8 gene expression restoration reduces c-myc oncogene expression in U937 cells. (A, B) Restoration of TDAG8 gene expression reduces c-myc oncogene expression at the mRNA level in U937 cells. (C) Over 14 days U937/TDAG8 GFP expression is reduced at physiological pH 7.4 while U937/Vector GFP is stable. (D) Reduction of U937/TDAG8 GFP expression is further augmented by activation of TDAG8 with acidic pH 6.9 treatment while the U937/Vector GFP is stable. ns: P > 0.05, *P < 0.05, ***P < 0.001. **Figure S3.** Restoration of TDAG8 gene expression in RPMI 8226 myeloma cells inhibits cell proliferation. (A) The empty vector does not substantially affect RPMI 8226 cell proliferation at physiological pH 7.4 in comparison to the RPMI 8226 parental cells. (B) Restoration of TDAG8 gene expression significantly reduces RPMI 8226 cell proliferation at physiological pH 7.4 in comparison to the RPMI 8226 parental cells. (C) The empty vector does not substantially affect RPMI 8226 cell proliferation at acidic pH 6.9 in comparison to the RPMI 8226 parental cells. (D) Restoration of TDAG8 gene expression significantly reduces RPMI 8226 cell growth at acidic pH 6.9 in comparison to the RPMI 8226 parental cells. ***P < 0.001. **Figure S4.** Restoration of TDAG8 gene expression increases apoptosis signaling. (A, B) Restoration of TDAG8 gene expression stimulates cleaved caspase 3 in U937 cells at physiological pH 7.4 and acidic pH 6.4. (A and C) Restoration of TDAG8 gene expression increases cleaved caspase 9 in U937 cells at physiological pH 7.4. (A and D) Restoration of TDAG8 gene expression increases cleaved PARP in U937 cells at acidic pH 6.4. ns: P > 0.05, *P < 0.05. **Figure S5.** Restoration of TDAG8 gene expression in Ramos lymphoma cells reduces primary tumor growth in SCID mice. (A) Representative image of a mouse subcutaneously injected with Ramos/Vector (left flank) and Ramos/TDAG8 (right flank) cells. (B) Restoration of TDAG8 in Ramos cells significantly delays primary tumor growth in SCID mice starting day 9 after injection. (C) Restoring TDAG8 gene expression in Ramos cells moderately reduces overall tumor mass after necropsy on day 21. (D) Representative image of Ramos/Vector and Ramos/TDAG8 tumors excised from SCID mice. ns: P > 0.05, *P < 0.05, **P < 0.01, ***P < 0.001. **Figure S6.** TDAG8 stimulates apoptotic signaling through Gα13/Rho signaling. (A, B) Inhibition of Gα13 signaling in U937/TDAG8 cells reduces cleaved caspase 3 and activation of Rho in U937/TDAG8 cells stimulates cleaved caspase 3. (A and C) Inhibition of Gα13 signaling in U937/TDAG8 cells reduces cleaved caspase 9 and activation of Rho in U937/TDAG8 cells stimulates cleaved caspase 9. (A and D) Inhibition of Gα13 signaling in U937/TDAG8 cells reduces cleaved PARP and activation of Rho in U937/TDAG8 cells stimulates cleaved PARP. ns: P > 0.05, *P < 0.05. **Figure S7.** Restoration of TDAG8 gene expression in U937 cells reduces attachment to a HUVEC monolayer and reduces migration toward a chemoattractant. (A) Restoration of TDAG8 gene expression reduces overall U937 cell attachment to a HUVEC monolayer while extracellular acidosis increases cell attachment. (B) Restoration of TDAG8 gene expression significantly reduces U937 cell migration toward a chemoattractant (SDF-1α) while extracellular acidosis reduces overall U937 cell migration. (C) In vivo imaging of a SCID mouse injected with U937/Vector-Luc cells that presented with hind limb paralysis and metastasis throughout the body. *P < 0.05, **P < 0.01, ***P < 0.001.

**Additional file 2: Table S1.** TDAG8 gene expression in various cancer types compared to normal tissues.

## Abbreviations

GPCR: G protein coupled receptor
Gα13: G protein alpha 13
pH: potential of hydrogen
RGS: regulator of G protein signaling
Rho: rho GTPase
RT-PCR: reverse transcription-polymerase chain reaction
SCID: severe combined immunodeficient
TDAG8: T cell death associated gene 8
