# Supplementary material for: Contextual tumor suppressor function of T cell death-associated gene 8 (TDAG8) in hematological malignancies
Source: J Transl Med. 2017 Oct 10;15:204. doi: 10.1186/s12967-017-1305-6 (PMC5634876; doi:10.1186/s12967-017-1305-6)

Additional Figure S1

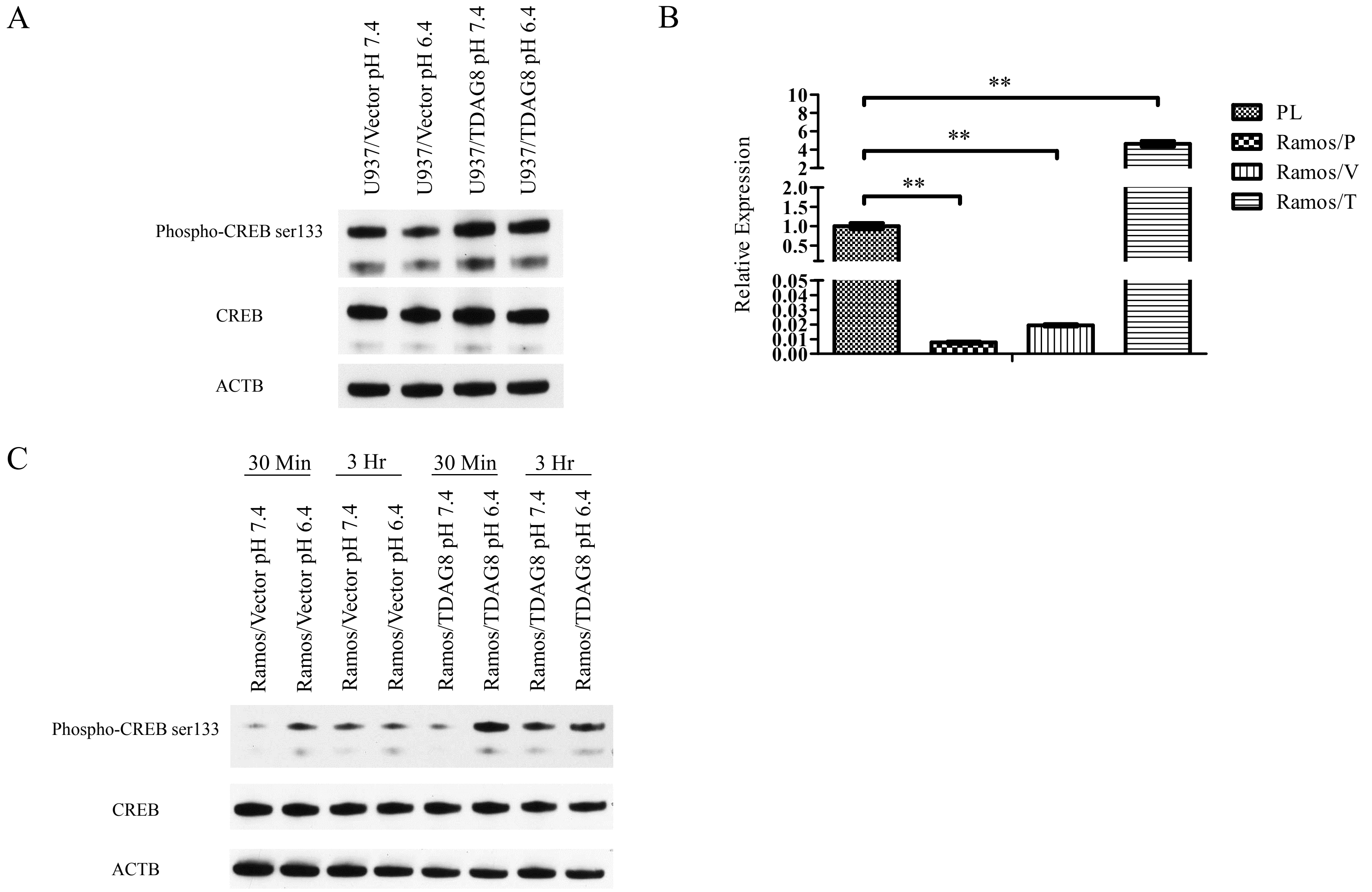

Additional Figure S2

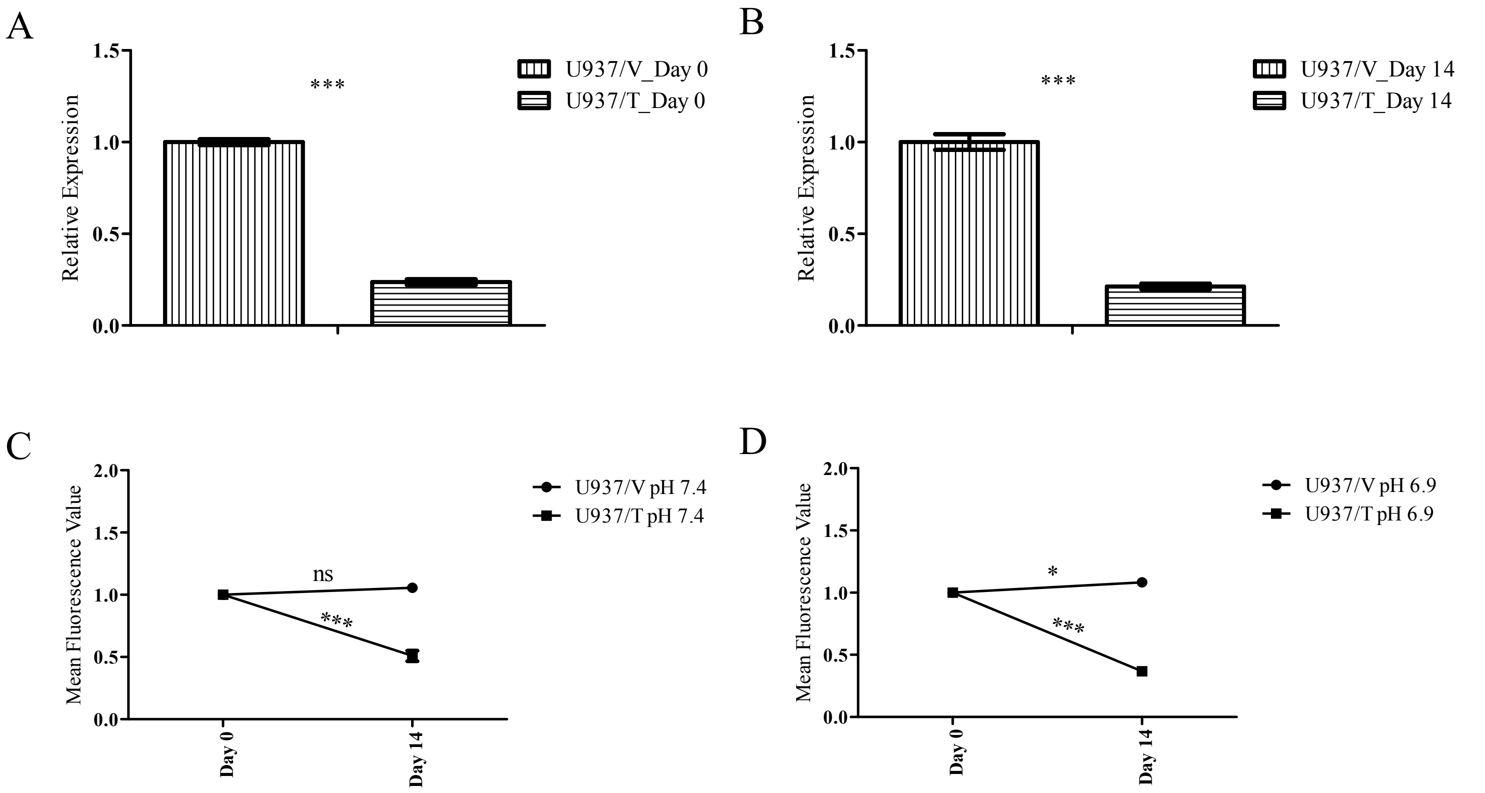

Additional Figure S3

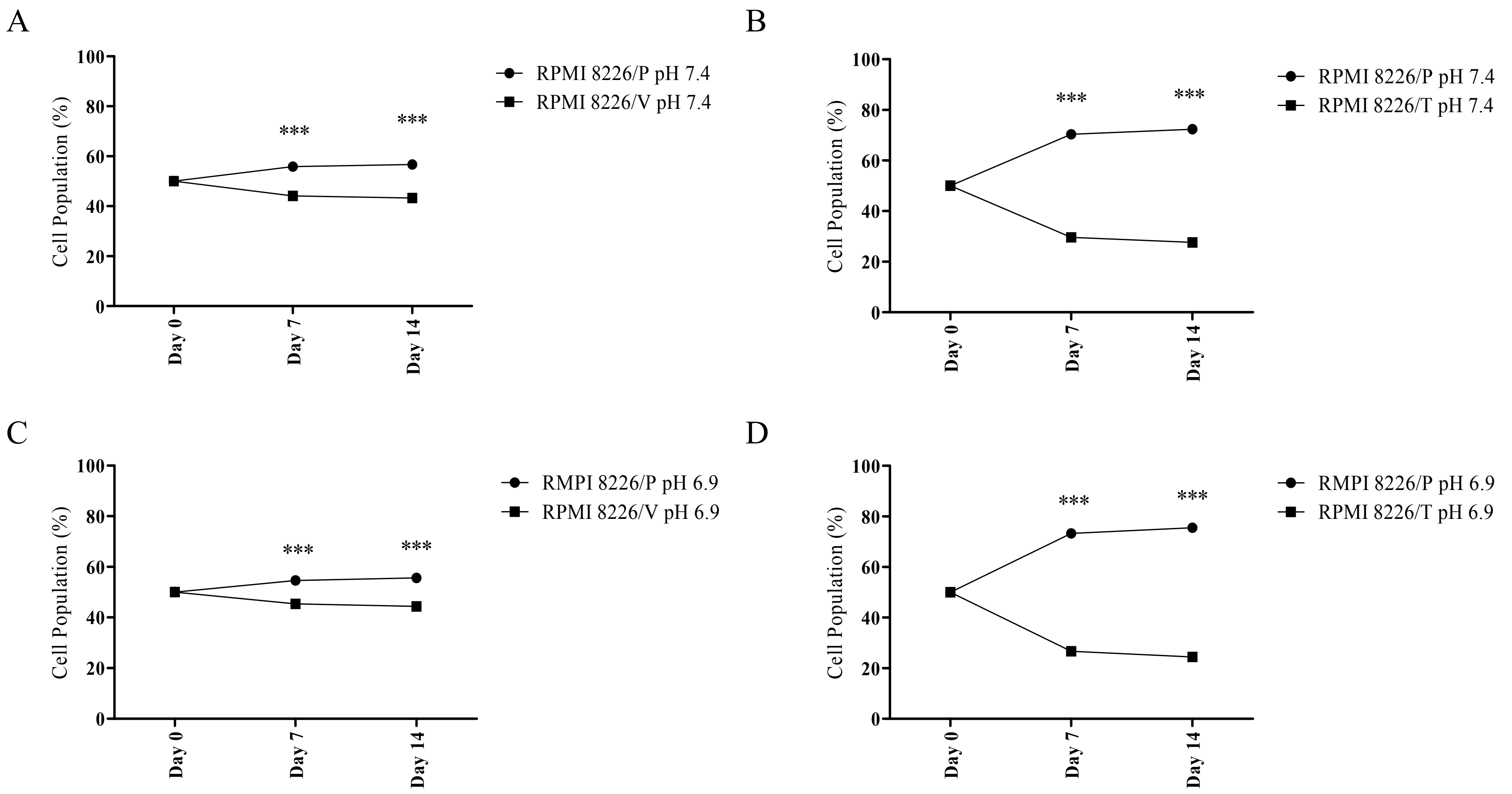

# Additional Figure S4

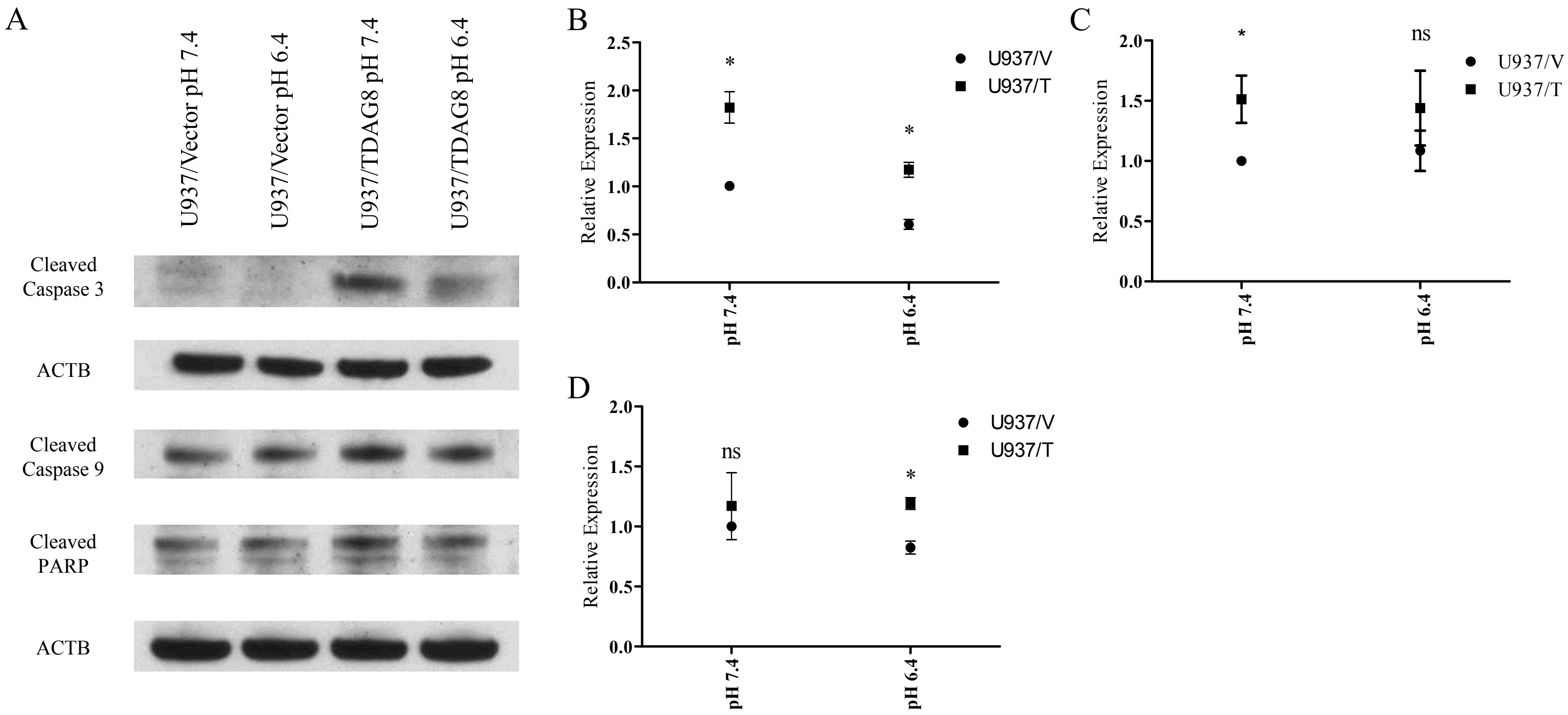

Additional Figure S5

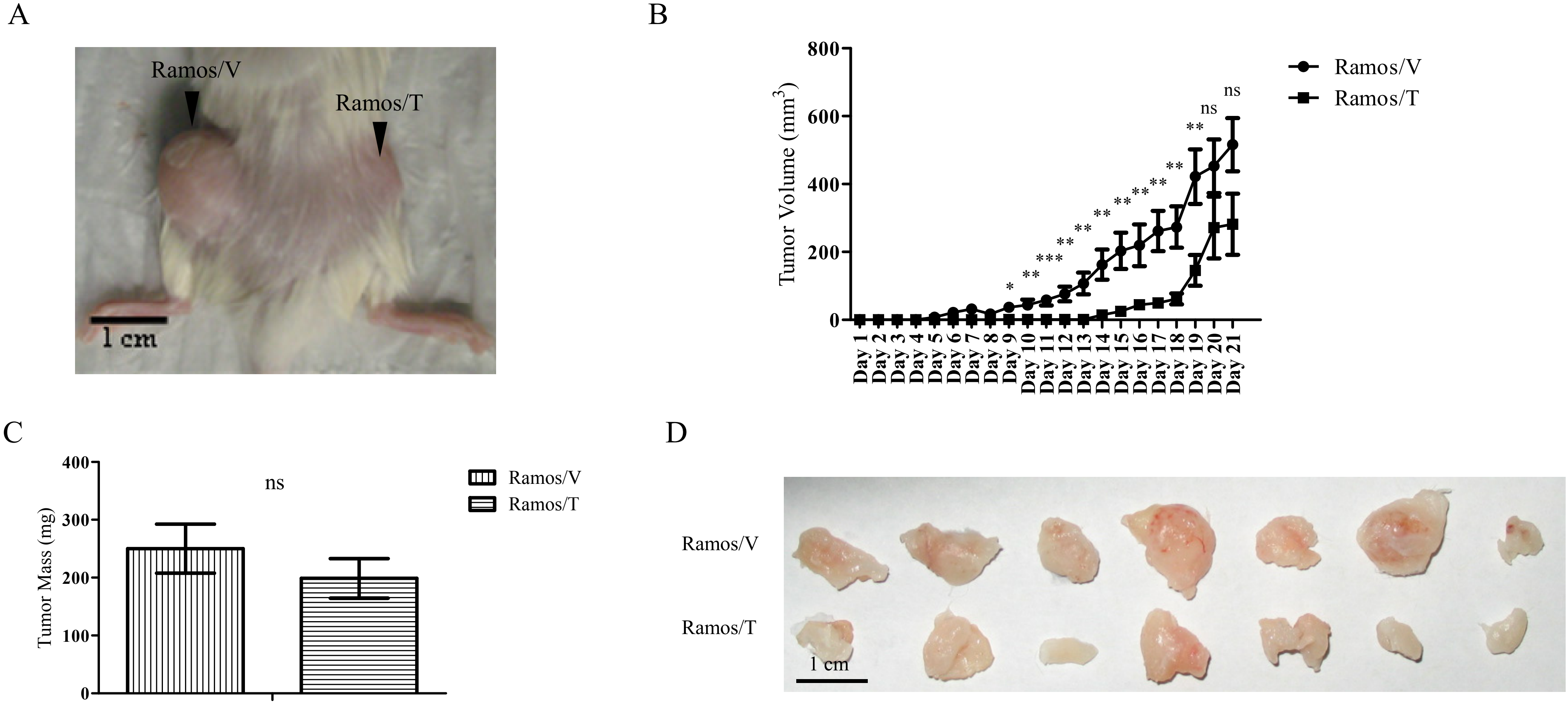

Additional Figure S6

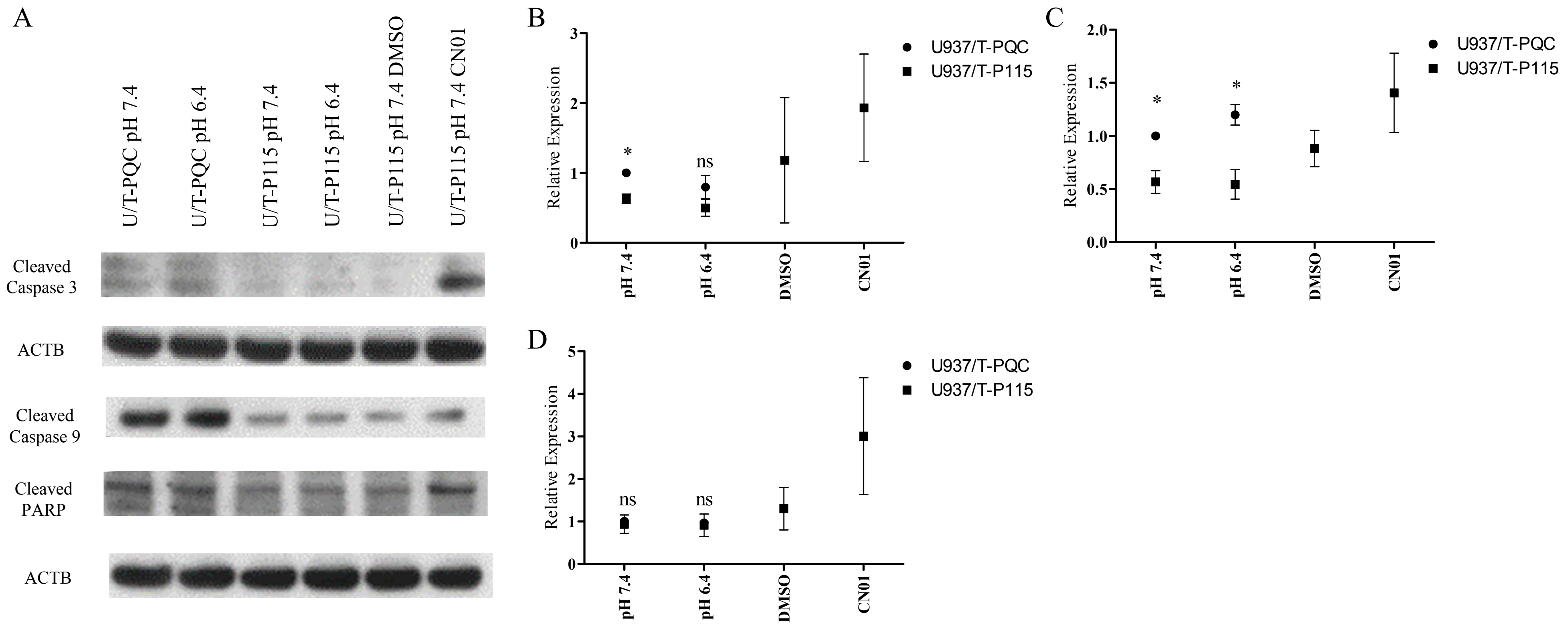

Additional Figure S7

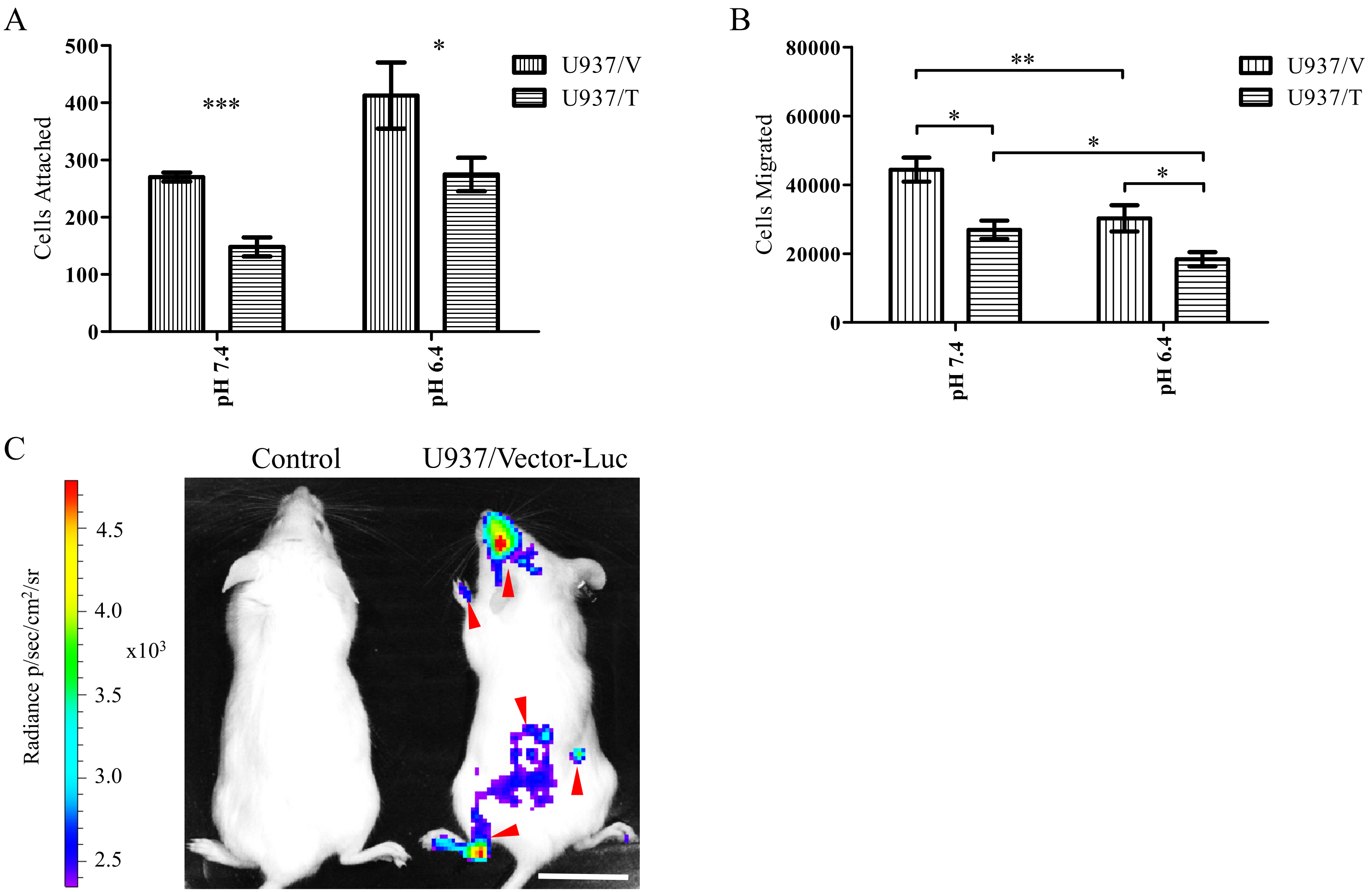

Supplement: Supplementary file 1 — Additional file 1: Figure S1. Restoration of TDAG8 gene expression results in increased phosphorylation of CREB at serine 133 in U937 and Ramos cells. (A) Restoration of TDAG8 gene expression in U937 cells results in stimulation of CREB phosphorylation at serine 133 indicating the TDAG8 activity level is increased. (B) TDAG8 gene expression is restored in Ramos cells to a level that is physiologically relevant. (C) Restoration of TDAG8 gene expression in Ramos cells results in stimulation of CREB phosphorylation at serine 133 indicating the TDAG8 activity level is increased. **P < 0.01. Figure S2. TDAG8 gene expression restoration reduces c-myc oncogene expression in U937 cells. (A, B) Restoration of TDAG8 gene expression reduces c-myc oncogene expression at the mRNA level in U937 cells. (C) Over 14 days U937/TDAG8 GFP expression is reduced at physiological pH 7.4 while U937/Vector GFP is stable. (D) Reduction of U937/TDAG8 GFP expression is further augmented by activation of TDAG8 with acidic pH 6.9 treatment while the U937/Vector GFP is stable. ns: P > 0.05, *P < 0.05, ***P < 0.001. Figure S3. Restoration of TDAG8 gene expression in RPMI 8226 myeloma cells inhibits cell proliferation. (A) The empty vector does not substantially affect RPMI 8226 cell proliferation at physiological pH 7.4 in comparison to the RPMI 8226 parental cells. (B) Restoration of TDAG8 gene expression significantly reduces RPMI 8226 cell proliferation at physiological pH 7.4 in comparison to the RPMI 8226 parental cells. (C) The empty vector does not substantially affect RPMI 8226 cell proliferation at acidic pH 6.9 in comparison to the RPMI 8226 parental cells. (D) Restoration of TDAG8 gene expression significantly reduces RPMI 8226 cell growth at acidic pH 6.9 in comparison to the RPMI 8226 parental cells. ***P < 0.001. Figure S4. Restoration of TDAG8 gene expression increases apoptosis signaling. (A, B) Restoration of TDAG8 gene expression stimulates cleaved caspase 3 in U937 cells at phys [file 12967_2017_1305_MOESM1_ESM.pdf]
