# Supplementary material for: Contextual tumor suppressor function of T cell death-associated gene 8 (TDAG8) in hematological malignancies
Source: J Transl Med. 2017 Oct 10;15:204. doi: 10.1186/s12967-017-1305-6 (PMC5634876; doi:10.1186/s12967-017-1305-6)
Supplement: Supplementary file 2 — Additional file 2: Table S1. TDAG8 gene expression in various cancer types compared to normal tissues. [file 12967_2017_1305_MOESM2_ESM.docx]

| Additional file 2: Table S1. TDAG8 Gene Expression in Various Cancer Types Compared to Normal Tissues | | | | | | | | |
| --- | --- | --- | --- | --- | --- | --- | --- | --- |
| Brain and CNS Cancer | | | | | | | | |
| Source | Sun, et al. | | | | TCGA | | | |
| Cancer | GBM | AA | | | GBM | | | |
| Fold Change | 3.406 | 2.221 | | | 3.924 | | | |
| P Value | 7.64E^-12^ | 9.03E^-5^ | | | 1.73E^-19^ | | | |
| N= | 81 | 19 | | | 542 | | | |
| Head and Neck Cancer Lung Cancer | | | | | | | | |
| Source | Ginos, et al. | | Hou, et al. | | Selamat et al. | Garber et al. |  | |
| Cancer | HNSCC | | LCLC | | LUAD | LUAD |  | |
| Fold Change | 3.307 | | -4.575 | | -1.372 | -2.388 |  | |
| P Value | 1.72E^-9^ | | 2.5E^-8^ | | 4.12E^-18^ | 0.009 |  | |
| N= | 41 | | 19 | | 58 | 39 |  | |
| **Kidney Cancer** | | | | | | |  | |
| Source | Jones, et al. | | Beroukhim, et al. | | Gumz, et al. | |  | |
| Cancer | CCRCC | UCC | | CCRCC | CCRCC | | |  |
| Fold Change | 6.158 | 5.154 | | 2.916 | 3.787 | | |  |
| P Value | 6.58E^-30^ | 3.08E^-12^ | | 5.20E^-9^ | 1.69E^-7^ | | |  |
| N= | 23 | 8 | | 27 | 10 | | |  |
| Glioblastoma (GBM), anaplastic astrocytoma (AA), Head and neck squamous cell carcinoma (HNSCC), Large-cell lung carcinoma (LCLC), Lung adenocarcinoma (LUAD), Clear cell renal cell carcinoma (CCRCC), and Urothelial cell carcinoma (UCC) tumors. Sun, et al. GMB and AA compared to normal brain (N=23). The Cancer Genome Atlas (TCGA), GMB compared to normal brain (N=10). Ginos et al. HNSCC compared to normal buccal mucosa (N=13). Hou et al. LCLC compared to normal lung (N=65). Selamat et al. LUAD compared to normal lung (N=58). Garber et al. LUAD compared to normal lung (N=5). Jones et al. CCRCC and UCC compared to normal kidney (N=23). Beroukhim et al. CCRCC compared to normal renal cortex (N=10) and renal tissue (N=1). Gumz et al. CCRCC compared to normal kidney (N=10). TDAG8 gene expression is increased in kidney, brain, and head and neck cancers when compared to normal control tissues. However, TDAG8 gene expression is reduced in lung cancers compared to normal lung tissues. | | | | | | | | |
